# Blood concentrations of a new psychoactive substance 4-chloromethcathinone (4-CMC) determined in 15 forensic cases

**DOI:** 10.1007/s11419-018-0427-8

**Published:** 2018-05-28

**Authors:** Ewa Tomczak, Mateusz Kacper Woźniak, Marzena Kata, Marek Wiergowski, Beata Szpiech, Marek Biziuk

**Affiliations:** 1 Department of Forensic Medicine, Faculty of Medicine, Medical University of Gdańsk, 3A Marii Skłodowskiej-Curie Str., 80-210 Gdańsk, Poland; 2 Department of Analytical Chemistry, Faculty of Chemistry, Gdańsk University of Technology, 11/12 Narutowicza Str., 80-233 Gdańsk, Poland

**Keywords:** 4-Chloromethcathinone, 4-CMC, Clephedrone, Blood concentration, Fatal and nonfatal intoxication, GC–MS

## Abstract

**Purpose:**

The 4-chloromethcathinone (4-CMC) is a synthetic derivative of cathinone and belongs to new psychoactive substances. Neither data on the effects of 4-CMC on the human body, nor on nontoxic, toxic and lethal concentrations in biological materials have been published in the literature. This paper describes the results of an analysis of the blood concentrations of 4-CMC determined in 15 forensic cases related to nonfatal intoxication including driving under the influence, and fatalities including overdoses, suicide and traffic accidents.

**Methods:**

A new method for the quantification of 4-CMC using gas chromatography–mass spectrometry (GC–MS) was developed. The symptoms of 4-CMC use were also studied based on an analysis of the documents prepared during the collection of samples or at autopsies.

**Results:**

The limits of detection and quantification of the method for blood samples were 0.3 and 1 ng/mL, respectively. The calibration curve was linear in the studied concentration range (1–500 ng/mL) with the correlation coefficient at 0.9979. The extraction recoveries varied in the range of 94.3–98.8%. The accuracy and precision were acceptable. The determined concentrations in nonfatal cases ranged from 1.3 to 75.3 ng/mL, and in fatalities from 56.2 to 1870 ng/mL.

**Conclusions:**

Our study can assist in the recognition of the possible effects caused by 4-CMC and can be helpful during the preparation of forensic toxicological opinions for courts of law. The validation parameters indicate the sensitivity and accuracy of the method. This is the first work presenting a validated method for the determination of 4-CMC in blood samples by GC–MS.

## Introduction

In the last decade, the black market for drugs has evolved enormously and offered both recreational and highly psychostimulating products. The report on Global Synthetic Drugs Assessment, published by the United Nations Office on Drugs and Crime (UNODC), stated that 348 new psychoactive substances (NPS) were identified between 2008 and 2013, while only 97 were identified in 2013 [1]. Since then, a total of 101 new substances were reported in 2014 for the first time to the EU Early Warning System (EWS) [2], while according to the “The World Drug Report” by the European Monitoring Centre for Drugs and Drug Addiction (EMCDDA) approximately 1 in 20 people between the ages of 15 and 64 used at least one drug in 2014 [3].

New psychoactive substances, such as synthetic cathinones and synthetic cannabinoids, remain the most commonly used drugs nowadays. They were introduced to illicit trade in 2006/2007 and are commonly sold under slang terms such as “legal highs”, “designer drugs”, “research chemicals”, “bath salts”, “plant food” or “room odorizers” [4–6]. They are usually produced in China and India, from where they are packed and exported to target countries, to be sold in the black market or via the Internet [7]. Because they are absolutely not designed for human consumption without any information on the dosage for safe use [8], they comprise a serious danger for human health.

**Fig. 1:**
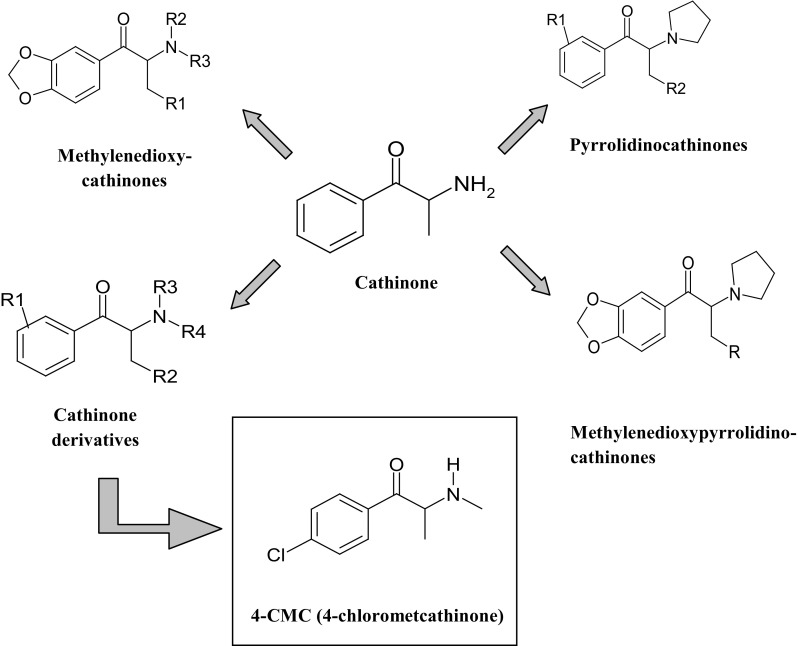
Structures of cathinone derivatives and 4-chloromethcathinone (4-CMC)

The 4-chloromethcathinone (1-(4-chlorophenyl)-2-(methylamino)-1-propanone), also known as 4-CMC or clephedrone, is a synthetic *p*-chlorine substituted derivative of cathinone and belongs to the NPS (Fig. 1). It has been commercially available on the black market since 2014 and is sold via the Internet in the form of white powder [4, 7]. The 4-CMC is currently a controlled drug in Germany, China and Virginia (USA), but until today it has not been classified as an illegal substance in the Act of Counteracting Drug Addition in Poland [9, 10]. Due to the novelty of the drug, there is no data in scientific literature considering the pharmacokinetics, pharmacodynamics, pharmacological and toxicological effects, and the potential of acute overdose effects and addiction in a long period of time. Wiergowski et al. [11] reported three cases of 4-CMC intake. However, these intoxication cases were considered also with a high dose administration of 25B-NBOMe. Therefore, the patients’ symptoms described by the authors were rather considered to be those by the 25B-NBOMe use, rather than by 4-CMC. Currently, the majority of information concerning the model of consumption of 4-CMC and its impact on the body originates from the anecdotal statements of users published on Internet forums [12]. Similarly to other synthetic cathinones, 4-CMC is typically snorted or ingested orally. Depending on the route of administration and the user’s tolerance, the dose of 4-CMC resulting in the desired effects ranges from 100 to 300 mg for oral ingestion and from 50 to 150 mg for snorting. Additionally, the effects of 4-CMC can be felt much faster after snorting than after oral ingestion (2–3 and 30–60 min, respectively), but they last for a shorter time. Also, the profile of effects of 4-CMC on the body depends on the route of administration; oral ingestion results in a euphoric effect, while snorting in a speed-like effect is accompanied by agitation, increased concentration, and increased self-confidence [12].

There are only two studies available in scientific literature describing procedures for the determination of 4-CMC by gas chromatography–mass spectrometry (GC–MS). Recently, Taschwer et al. [4] characterized 4-CMC as an NPS by the analysis of crystals, which were bought via the Internet, and presented its spectrum in electron ionization (EI) mode [4]. In another study, Klavz et al. [7] developed a method for the quantification of 4-CMC and four other NPS in seized materials (powders) found at a scene, and also in human urine and stomach contents. However, 4-CMC was not detected in the biological materials investigated [7]. Therefore, to the best of our knowledge, there is a lack of accurate studies concerning reference concentrations of 4-CMC in biological materials in particular types of cases. Therefore, the aim of this study was to present and summarize the results of an analysis of 15 cases in which 4-CMC was detected and quantified in blood. This paper also discusses the concentrations of 4-CMC in different types of forensic cases related mainly to driving under the influence (DUI) and traffic accidents, as well as fatalities including overdoses and suicides. The results of our study can assist in the recognition of possible effects caused by this drug and can be helpful during the preparation of reports of toxicology analysis for courts of law for proper data interpretation. A validation of the new methodology developed for 4-CMC determination in blood by GC–MS in electron ionization mode is also presented.

## Materials and methods

### Standards and chemicals

A certified standard solution of 4-CMC monohydrochloride in methanol at a concentration of 1 mg/mL (as a free base) was manufactured by Cayman Chemicals (Ann Arbor, MI, USA); a methanolic solution of racemic-methamphetamine-*d*_*5*_ (*rac*-mAMP-*d*_*5*_) at a concentration of 0.1 mg/mL was manufactured by LGC Standards (London, UK). Both solutions were delivered by a subdivision of LGC Standards (Dziekanów Leśny, Poland).

Acetonitrile (HPLC grade purity) was purchased from Merck Millipore (Warsaw, Poland); 1-chlorobutane (HPLC grade) and pentafluoropropionic anhydride (PFPA) (99% purity) from Sigma-Aldrich (Warsaw, Poland); hydrochloric acid (HCl) at a concentration of 35–38%, potassium carbonate (K_2_CO_3_) and sodium chloride (NaCl) powder (all analytical grade) from POCH (Gliwice, Poland). Ultrapure water was produced by a Millipore Synergy 185 ultra-pure water system (Millipore, Warsaw, Poland).

### Blood samples

Blood samples were sent to the Department of Forensic Medicine, Gdańsk, Poland or collected during an ongoing autopsy. The provision documents from investigative authorities to carry out routine toxicological analyses for the presence of psychoactive substances and prescription drugs were also provided. In a number of cases, there were high suspicions of drug administration, revealed by observations of patients’ behaviours during the police control or medical interview before blood collection. These documents were attached to most of the collected samples, which allowed us to discuss some symptoms of 4-CMC use. Preliminary toxicology analyses for common drugs of abuse and prescription drugs, being routinely performed in our laboratory, involved the use of an enzyme-linked immunosorbent assay, headspace gas chromatography–flame ionization detection, high-performance liquid chromatography–diode array detection and GC–MS with electron/chemical ionization. Samples positive for 4-CMC were quantitatively analysed by the method developed in this study and described below.

The blood used as blank samples for the development and validation of the method was obtained from a regional blood donation centre (Gdańsk, Poland) and was stored in − 20 °C prior to analysis. Blank blood was screened to be negative for drugs of abuse (including 4-CMC).

### Preparation of stock solutions, calibrators and quality control samples

Stock solutions of 4-CMC were prepared in methanol by diluting the certified standard solution to reach concentrations at 50 and 5 µg/mL. The purchased standard solution of mAMP-*d*_*5*_ was used as the stock solution of the internal standard (IS). All solutions were used for calibration and validation, and were stored at − 20 °C prior to analysis.

The calibrators (*n *= 3) were prepared by spiking 1 mL of blank blood samples with an appropriate amount of the stock solution to obtain the concentrations of 1, 5, 10, 25, 50, 100, 250, and 500 ng/mL. The concentration of the IS in each calibrator was maintained at 500 ng/mL by adding 5 µL of the IS stock solution. Finally, the extraction and derivatization procedure was performed.

Quality control (QC) samples were prepared in triplicate at three concentration levels within the range of concentrations of calibration solutions: low—5 (LQC), medium—100 (MQC) and high 500 (HQC) ng/mL. QC samples were used for the evaluation of the repeatability and recoveries of the method.

Samples with concentrations higher than 500 ng/mL were diluted with drug-free blood to fit into the linear range to allow quantification.

### Extraction

To a blood sample (1 mL) placed in a 15 mL screw-capped glass tube, 5 μL of the IS solution was added followed by 2 mL of 5 M K_2_CO_3_ solution and 2 mL of saturated NaCl solution. Protein precipitation was performed with 2 mL of acetonitrile and by mixing the sample for 1 min. Subsequently, after the addition of 2 mL of 1-chlorobutane, mechanical stirring was carried out for 2 min. Finally, the sample was centrifuged for 3 min at 3500 rpm, and the organic layer was transferred to a clean glass tube. Then, an additional 2 mL of 1-chlorobutane was added to the blood residuals after the first extraction, and the sample was vortex-mixed for 2 min and re-centrifuged for 3 min at 3500 rpm. The organic layer was combined with the first extract. After the addition of 100 µL of HCl solution in methanol (1:9, v/v), the extraction solvent was evaporated to dryness under a gentle stream of nitrogen at 40 °C, and the dry residue was dissolved in 50 μL of ethyl acetate. Then, 50 µL of PFPA was added to perform derivatization (20 min, 55 °C). Finally, the solution was evaporated to dryness and the dry residue was dissolved in 50 µL of ethyl acetate. A 2-µL aliquot was injected into the GC–MS apparatus.

### GC–MS conditions

All analyses were performed using a 7890A GC System (gas chromatograph) equipped with a G4567A autosampler and a split/splitless injection port, and connected with a 5975C single quadrupole mass spectrometer (MS) with an electron ionization ion source (all Agilent Technologies, Santa Clara, CA, USA). The separation of analytes was carried out on a Phenomenex ZB-5 MS capillary column (30 m × 0.25 mm i.d., 0.25 µm film thickness; Phenomenex, Izabelin, Poland) with helium at a purity of 99.999% as the carrier gas at a constant flow of 1 mL/min. The split injection mode (10:1) was used. The oven temperature was programmed at 50 °C for 1 min, then increased to 160 °C at 30 °C/min, finally ramped up to 250 °C at 5 °C/min and held for 1 min. The temperatures of the injection port, MS transfer line, ion source and detector were set at 280, 285, 280 and 200 °C, respectively. The MS was operated in positive mode (electron energy 70 eV). Full-scan acquisition was performed with the mass detection range set at *m/z* 40–380. For the identification and quantification of the derivatized analyte/IS, selected ion monitoring (SIM) mode was used with the ions at *m/z* **204**, 139, 160 for 4-CMC and **208**, 163, 119 for the IS. The bolded ions were used for quantification. All the ions were chosen due to their specificity and abundance. Data acquisition and analysis were accomplished with MSD ChemStation software by Agilent Technologies (version E.02.02.1431).

### Method validation

The developed GC–MS-based method for 4-CMC quantification was validated according to international guidelines in the field of our study [13, 14] in terms of selectivity, linearity, sensitivity, limit of detection (LOD) and limit of quantification (LOQ), matrix effect, carry-over, recovery and repeatability.

The selectivity experiments were performed to verify the presence of endogenous or exogenous compounds at the retention time of the analyte or the IS. For this purpose, ten blood samples obtained from different subjects who are not involved in drugs of abuse were carefully checked by our GC–MS-based screening method after the extraction/derivatization procedure.

To compensate the variability of the detector signal during different analyses and losses of analyte in the extraction and derivatization step (correction of recovery), the IS calibration was performed. An eight-point calibration curve was constructed using the peak area ratio (4-CMC vs. IS) plotted against the concentration (the number of replicates for each level, *n *= 3). In order to increase the accuracy at the low concentration levels, the weighing factor of 1/*x*^2^ was applied to the calibration curve. The linearity of the weighted calibration curve was evaluated in the range of 1–500 ng/mL of blood and was expressed as the correlation coefficient (*r*). The LOD of the method was assessed using the MSD ChemStation software and calculated (by extrapolation) based on the analysis of samples (*n *= 3) at the lowest concentration level from the calibration curve (1 ng/mL). The signal-to-noise ratio equal to 3 reported for the least abundant among qualifier ions was considered as LOD. The lowest point of the calibration curve was assumed as the LOQ.

The matrix effect (ME) of the developed method was evaluated by comparing the slopes of the eight-point calibration curves (*n *= 3) prepared in extracts obtained from drug-free blood with those prepared in a solution (methanol) and calculated using the following equation [15]:

$${\text{ME }}(\% ) = \left( {\frac{{{\text{slope }}\;{\text{of}}\;{\text{calibration }}\;{\text{curve}}\; {\text{in}}\;{\text{matrix}}}}{{{\text{slope}}\;{\text{of }}\;{\text{calibration}}\; {\text{curve}}\; {\text{in}}\; {\text{solvent}}}} - 1} \right) \times 100\% .$$

The potential for carry-over of the analyte and the IS to the subsequent sample in the autosampler sequence was evaluated by injecting 2 µL of ethyl acetate solution after analysis of blank blood samples fortified with the IS and the analyte at the highest concentration level from the calibration curve (500 ng/mL). The test was performed in six replicates.

The extraction recoveries (in %) were determined by comparing the analyte-to-IS peak area ratios for the spiked (analyte only without IS) and extracted/derivatized blank blood samples according to the above procedure with the corresponding analyte-to-IS peak area ratios of the matrix extracts fortified with the reference standard after the extraction (just before derivatization) at concentrations of QC samples (*n *= 3 each). In this test, the IS was exclusively added after extraction (just before derivatization). The repeatability of the method was estimated as intra- and interassay accuracy and precision. Intraday assay experiments were carried out by analysing QC samples (*n *= 3). To evaluate the interday assay repeatability (as between-day averages), the tests were repeated over three consecutive days. The accuracy of the method was calculated as the mean ratio of the measured and the nominal concentrations, while precision was assessed as percent coefficients of variation (% CVs) of these measurements.

## Results and discussion

### GC–MS and validation

**Fig. 2:**
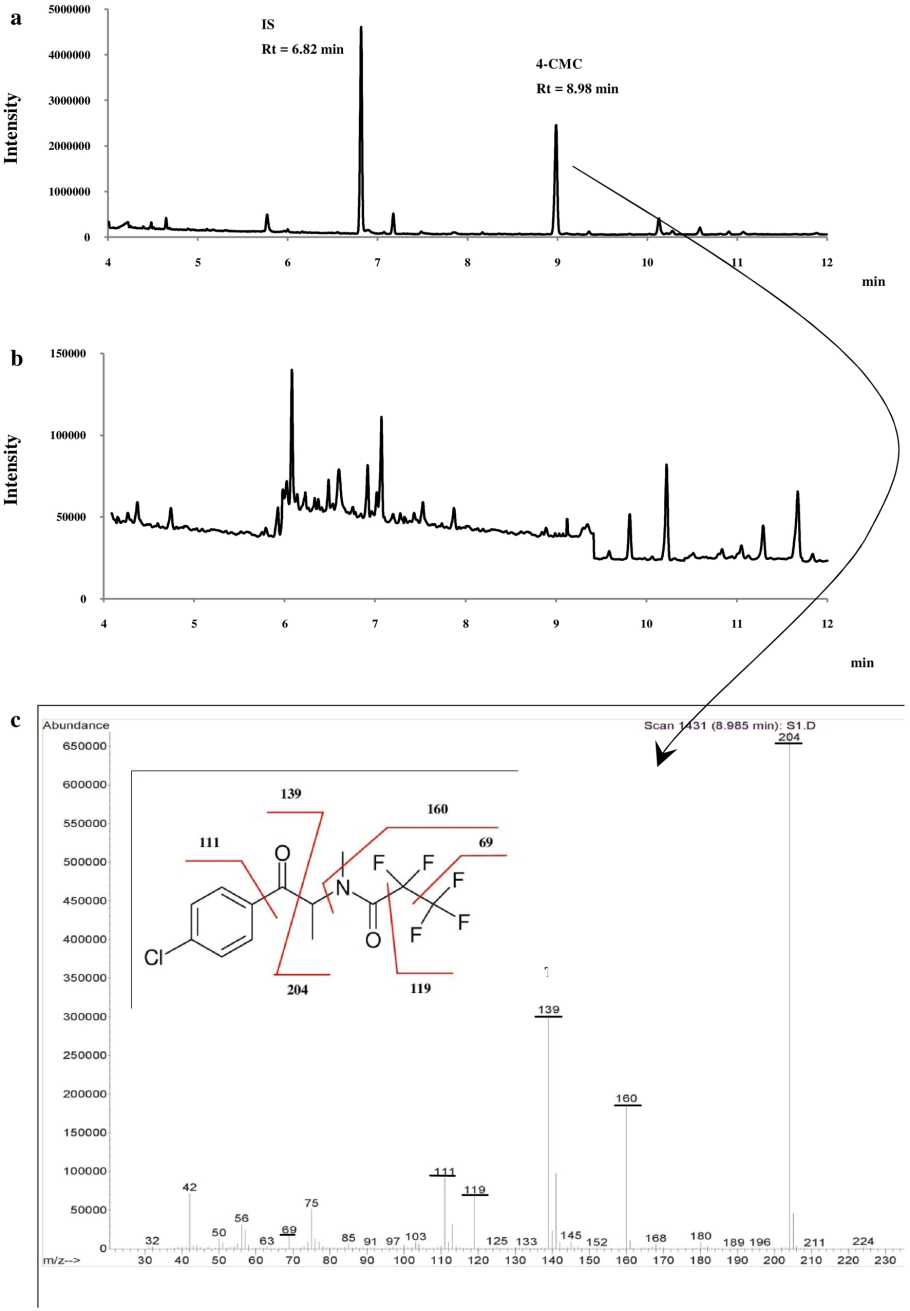
**a** Total ion current chromatogram of a mixture of derivatized 4-CMC and internal standard (IS) at a concentration of 50 µg/mL, **b** selected ion monitoring chromatogram for drug-free blood sample at *m/z* 204 for the selectivity test, and **c** mass spectrum plus proposed fragmentation pattern of derivatized 4-CMC. *Rt* retention time

The developed method includes three ions (one quantifier and two qualifiers) for the determination of 4-CMC in blood samples. The total ion current chromatogram of 4-CMC and the IS prepared in methanol at a concentration of 50 µg/mL after derivatization with PFPA is presented in Fig. 2a. A fragmentation pattern of derivatized 4-CMC was also suggested (Fig. 2c). The increased sensitivity and the better signal-to-noise ratio was enabled by careful optimization of chromatographic conditions, such as the temperature of the injector, the initial and final column temperatures, the temperature ramping up rate and carrier gas flow rate, as well as the temperature elements of the MS.

No interfering peaks due to endogenous/exogenous substances that could have obstructed the identification and quantification of the compounds of interest were observed in the drug-free samples investigated for selectivity at the retention times of the analyte and the IS (Fig. 2b). Therefore, the developed method can be considered as highly specific and selective for the determination of 4-CMC. The data of the parameters of calibration curves to establish the ME are summarized in Table 1. A negative value of the matrix effect was obtained, which indicates the suppression of the detector signal by co-extracted compounds as compared to the signal of the analyte injected in the solvent. However, MEs in the range of − 20 to + 20% are perceived as soft and can be neglected [15]. A carry-over effect was not observed. The method was shown to be linear within the tested range (1–500 ng/mL). The correlation coefficient (*r*) of the weighted calibration curve was 0.9979. The LOD and LOQ of the method were 0.3 and 1 ng/mL, respectively. The accuracy, precision and recoveries data for intra- and inter-day measurements are summarized in Table 2.

**Table 1:** Regression equations for neat spiked solvent and spiked blood extract for assessment of matrix effect (ME) of the proposed method (eight point calibration curves, *n *= 3) for quantification of 4-chloromethcathinone (4-CMC)

Matrix	Curve’s equation	*r*	ME (%)
Solvent	*y* = 0.00233*x* − 0.00048	0.9993	− 11.2
Blood extract	*y* = 0.00207*x* − 0.0010	0.9996	

*r* correlation coefficient

**Table 2:** Validation parameters of the method: accuracies, precisions and recovery rates (%) (*n *= 3) for quantification of 4-CMC

Concentration (ng/mL)	Intraday assay (%)			Interday assay (%)	Recovery (%) (mean ± SD)
	Day 1	Day 2	Day 3		
5	96.8 (0.3)	102 (0.6)	92.7 (0.7)	97.3 (4.4)	98.2 ± 2.5
100	95.3 (3.6)	99.9 (5.6)	102 (1.1)	98.9 (5.2)	98.8 ± 3.1
500	96.1 (1.8)	94.8 (1.4)	98.7 (1.2)	96.5 (2.2)	94.3 ± 4.1

The precision expressed as percent coefficient of variation is shown in parenthesis

*SD* standard deviation

All the validation parameters fulfilled the established international criteria for bioanalytical methods. Therefore, the developed method for the determination of 4-CMC in blood samples is characterized by high accuracy and precision and can be used for the analysis of real samples. Moreover, the low LOD and LOQ demonstrate that our method is sensitive and is well suited for the quantification of the blood concentration of 4-CMC in forensic cases at even small concentrations of this drug.

### Toxicological cases

In Poland, the popularity of 4-CMC as an NPS has increased year by year. In the first half of 2015, it was the eighth psychoactive substance most frequently found in “designer drugs”, and in the second half of 2015, it ranked second. Taking into account the data for 2016, 4-CMC was the psychoactive substance most frequently found in these types of products [16]. At the time when this article was written, it was not yet under legal control in Poland.

The cases (*n *= 15) in which 4-CMC was detected, were analysed between 2015 and 2017 (Table 3). In 93.3% of cases of 4-CMC users, there were mostly males (14 cases), and only a single case was a woman (6.7%). Other authors described a similar gender scheme for synthetic cathinones [17]. The age range for the cases under study was 18–38 years (*n *= 12; mean 24.5 years; median 23.5 years). Therefore, young men are the main users of drugs, which is comparable to data from relevant literature [5, 17].

**Table 3:** Fatal and nonfatal intoxication cases involving 4-CMC

Case number	Sex/age (years)	Symptoms	Type of case/case description	4-CMC blood concentration (ng/mL)	Other drug blood concentration (ng/mL; alcohol in g/L)
Nonfatal case					
1	M/35	Slurred speech, slowed behaviour, drowsiness, dilated pupils, disorientation as to time, “obligatory body posture”	DUI	2.0	Diazepam: 14.3
2	M/23	Facial redness, tachycardia	DUI	7.9	MDMA: 755 MDA: 50 Ethyl alcohol: 1.29
3	M/20	No abnormalities discovered	Road accident (driver)	33.4	THC: < LOQ THC-COOH: 37
4	M/U	Slurred speech, eye redness, facial redness	DUI	25.4	Amphetamine: 15 3-MMC: 450 THC-COOH: 8
5	M/24	Markedly agitated and distracted, logorrhea, pupils unreacted to light	DUI and drug possession	75.3	Estazolam: 8.1 Nordazepam: 28.8 Diazepam: 14.4
6	M/26	The patient was hospitalized: he was restrained while being transported to the hospital. The patient was joyful, and exhibited increased drive, tachycardia, dilated pupils, and difficulty in picking up objects from the ground	DUI	5.5	–
7	M/25	Slurred speech, dilated pupils with poor light reflex, difficulty in walking	DUI	27.4	Amphetamine: 249
8	M/20	Dilated pupils with poor light reflex. Difficulty in picking up objects from the ground. Positive Romberg’s sign	Burglary	11.8	THC: < LOQ THC-COOH: 15
9	M/18	Dilated pupils with poor light reflex. Disturbed orientation as to time, place and surroundings	DUI	1.3	Amphetamine: 9.8 BZE: 297 EME: 23
Fatal case					
10	M/20	n/a	Death in road accident (passenger)	183	THC: 13.5 THC-COOH: 160 Ethyl alcohol: 0.67
11	M/20	n/a	Death in road accident (passenger)	78.4	THC: 3.8 THC-COOH: 57 Ethyl alcohol: 0.72
12	M/U	n/a	Death in train accident - suicide	56.2	Ethyl alcohol: 2.68
13	M/25	n/a	Body found in an apartment	394	Amphetamine: 2200
14	M/38	n/a	Body found in an apartment	698	Nordazepam: 308
15	F/U	n/a	Fall from a height - suicide	1870	Amphetamine: 861 THC: 12 THC-COOH: 85

*DUI* driving under the influence, *n/a* not available, *U* unknown, *3*-*MMC* 3-methylmethcathinone (3-mephedrone), *BZE* benzoylecgonine, *EME* ecgonine methyl ester, *THC* tetrahydrocannabinol*, THC*-*COOH* 11-nor-9-carboxy-tetrahydrocannabinol, *LOQ* limit of quantification

In a just single case (No. 6), 4-CMC was determined as the only psychoactive substance, and in the other cases, other pharmacologically active substances and/or their metabolites were also detected. The most frequent combination found in our study was the combination with THC/THC-COOH (*n *= 6). The combination of another synthetic cathinone (α-PVP) with cannabinoids was also reported as the most popular combination in other studies [17]. The other determined compounds included amphetamine (*n *= 5), ethyl alcohol (*n *= 4), benzodiazepines (*n *= 3, diazepam, nordazepam, and estazolam), MDMA (*n *= 1), MDA (*n *= 1), 3-MMC (*n *= 1), and cocaine metabolites (*n *= 1, ecgonine methyl ester and benzoylecgonine). This indicates that young people have a tendency to use several psychoactive substances, often marked by a profile of similar effects, at the same time. This may lead to drug-drug interactions (DDI), including life- or health-threatening additive or superadditive synergism, and, in consequence, some effects which could not be foreseen by the users [18, 19]. Such a situation may take place, for example, in the case of the simultaneous use of synthetic cathinones (including 4-CMC) and amphetamine-type stimulants, since both classes of substances are essentially phenethylamine derivatives. The substances have a similar mechanism of action; they inhibit monoamine transporters, which are responsible for the reuptake of monoamines in the synaptic cleft of the neurons [20]. This may explain the symptoms observed in case 9, clearly indicating the use of psychoactive substances, despite the detection of relatively low concentrations of 4-CMC and amphetamine.

### Nonfatal cases

For nonfatal cases (1–9), the concentrations of 4-CMC in blood ranged between 1.3 and 75.3 ng/mL (*n *= 9; mean concentration 21.1 ng/mL; median 11.8 ng/mL). These cases mainly concerned drivers stopped upon traffic control due to the suspected consumption of alcohol or psychoactive substances (driving under the influence, DUI; 77.8%, *n *= 7), traffic accident (11.1%, *n *= 1) or burglary (11.1%, *n *= 1).

Among DUI, the concentration of 4-CMC ranged from 1.3 to 75.3 ng/mL (mean 22.3 ng/mL; median 16.7 ng/mL). In the case of a burglary, 11.8 ng/mL of 4-CMC was determined. In the case of the driver who caused a fatal accident involving two passengers (driver: case 3; passengers: cases 10 and 11), the concentration of 4-CMC amounted to 33.4 ng/mL. It was the only case with no symptoms indicating effects of psychoactive substances. In the other eight nonfatal cases, symptoms indicating the consumption of alcohol or ingestion of psychoactive substances were identified during traffic control and/or medical examination. The most frequently observed disturbances of psychomotor functions in the cases under analysis included dilated pupils (*n *= 5), slow or absent pupillary light reflex (*n *= 4), slurred speech (*n *= 2), agitation, increased drive (*n *= 2), tachycardia (*n *= 2), difficulty in walking (*n *= 2), difficulty in picking up objects from the ground (*n *= 2), positive Romberg’s sign (*n *= 1), disorientation as to time, place, and surroundings (*n *= 1), drowsiness (*n *= 1), logorrhea (*n *= 1), and slowed behaviour (*n *= 1). Enforced body position was applied in two cases (nos. 1 and 6).

Beside 4-CMC, psychostimulants and/or their metabolites, including amphetamine, MDMA, MDA and 3-MMC as well as ecgonine methyl ester and benzoylecgonine were also determined in four cases under study (cases 2, 4, 7 and 9). The determination of the relatively high concentrations of the aforementioned stimulants in blood, along with 4-CMC (cases 2, 4 and 7), as well as the clearly observable symptoms of intoxication, did not make it possible for the authors to recognize 4-CMC as the only substance responsible for the symptoms. In case 9, despite the effects on the central nervous system observed during medical examination, relatively low concentrations of amphetamine and 4-CMC as well as the presence of psychologically inactive cocaine metabolites were also detected. This is puzzling, but we may suspect that prior to analysis, cocaine in blood underwent degradation in vitro as a result of the improper storage of biological material before its delivery to the laboratory.

In just a single case (case 6), only 4-CMC was detected. However, the concentration of 4-CMC in the blood (5.5 ng/mL) was several times lower than the mean concentration, but we should take into account that as many as 3 h had passed between the car journey and the collection of the blood sample. The concentration of 4-CMC may have been considerably higher when the patient was in the car. The subject was agitated, displayed increased drive, and, according to a witness, behaved “like a monkey in a cage” or as if he had experienced an epileptic seizure. Additionally, the pupils of this driver were dilated and tachycardia was detected. The patient was restrained with belts during transportation from the place in which he was stopped by traffic control to the hospital. However, the abovementioned symptoms could not be clearly identified as specific to 4-CMC, because in the other eight cases, all patients took at least one psychoactive substance except 4-CMC. In case 5, beside 4-CMC, benzodiazepines (diazepam, nordazepam and estazolam) were also quantified. Diazepam and nordazepam are frequently used for patient sedation and these medicines might have been administered by the medical staff. Estazolam is not administered for medical purposes in Poland, and, therefore, it must have been taken for abusing purposes. However, this has not been confirmed by the investigating authorities (no relevant packaging was found next to the patient, and he did not confirm having taken estazolam). Furthermore, the symptoms observed in this case were typical of psychostimulants (marked agitation, logorrhea, no pupillary light reflex), and the concentration of 4-CMC was highest of all nonfatal cases under analysis. Diazepam was also determined in the blood in case 1, but it was most probably administered during medical intervention.

### Fatal cases

In the majority of fatal cases (*n *= 5), the concentration of 4-CMC was markedly higher than in nonfatal ones (56.2–1870 ng/mL; mean concentration 547 ng/mL, median 288 ng/mL). However, four of the fatal cases were connected with events possibly resulting from risky behaviour being a typical effect of a class of stimulants. In the cases of deaths resulting from car accidents, 4-CMC concentrations amounted to 78.4 and 183 ng/mL (cases 10 and 11), respectively. THC, THCCOOH and ethyl alcohol were also detected in these cases. Furthermore, ethyl alcohol and cannabinoids plus amphetamine were also quantified in two suicidal death cases (12 and 15). Moreover, two cases (13 and 14) may be identified with acute intoxication with psychoactive substances (including 4-CMC), and this hypothesis was also suggested by forensic pathologists.

Case 13 concerns a 25-year-old man who was found dead in his flat. The autopsy revealed that the immediate cause of death was acute cardiac failure in the mechanism of arrhythmias due to chronic focal lesions in the myocardium. Moreover, according to the forensic pathologist, considering the pathomechanism of his death, the acute cardiac failure does not contradict the possibility of an unknown substance being co-responsible for the intoxication. This is suggested by the presence of gastric contents in his respiratory tract as a result of vomiting. Therefore, this case may be considered death resulting from a mixed causes of myocardium lesions with 4-CMC and amphetamine intoxication, as the concentrations determined in his blood (394 and 2200 ng/mL, respectively) are relatively high, and the detected concentration of amphetamine can be fatal to a nonaddict [21].

Case 14 concerns a 38-year-old man who was found dead in his flat. During autopsy, blood and a fragment of abdominal wall containing a pill were collected for toxicological tests. Analyses showed that the pill contained disulfiram, although it was not detected in the blood. Ethyl alcohol was not detected either. The autopsy did not reveal any bodily injuries. However, signs of sudden death were found, including recent passive congestion of internal organs, presence of liquid blood in the heart and great vessels, and intensive livor mortis. Moreover, the forensic pathologist indicated the action of a psychoactive substance on the body probably as the primary cause of his death. Therefore, it is highly probable that the death resulted from an overdose of 4-CMC only, because only nordazepam at therapeutic concentration was additionally determined.

Case 15 concerns a suicidal death of a young woman caused by a fall from a height. What is noteworthy, a much higher concentration of 4-CMC was determined in the blood in this case than in the case of deaths caused by acute intoxication with psychoactive substances (cases 13 and 14). Therefore, the determined concentration of 4-CMC at a level many times higher than in the other cases of fatal intoxications presented may be an argument in favor of the probable addictive effect of 4-CMC, and her body might have developed tolerance to this substance, resulting in the users increasing the taken doses of the substance. This effect is observed for the majority of amphetamine-type stimulants.

## Conclusions

The 4-CMC is a psychostimulating substance very dangerous for health but not banned in many countries, including Poland. Intoxication cases with NPS and their unpredictable effects are very difficult to recognize, which makes these drugs an important health issue, especially when other drugs are used in combination. This is an effect of a rapid evolution in the availability of NPS and a large number of their possible structures. Therefore, there is still an urgent need for the development of new methods for the determination of NPS in various biological materials. The results can be useful for the proper interpretation of toxicological results. To the best of the authors’ knowledge, this is the first work presenting blood reference concentrations of 4-CMC in different toxicological cases and a validated method for the determination of this drug in blood samples by GC–MS. Finally, our developed method can be an alternative for most toxicological laboratories, which do not include a liquid chromatography–tandem mass spectrometry system, which is usually used for NPS analysis nowadays.
